# Using technology to increase reach and optimize consent experience for a large-scale research program

**DOI:** 10.1017/cts.2024.640

**Published:** 2025-01-25

**Authors:** Emma Coen, Daniel P. Judge, Samantha Norman, John T. Clark, Andrew Cates, Randolph Thornhill, Kelly Hunt, Lori McMahon, Leslie Lenert, Caitlin G. Allen

**Affiliations:** 1 Department of Public Health Sciences, Medical University of South Carolina, Charleston, SC, USA; 2 Department of Medicine, Medical University of South Carolina, Charleston, SC, USA; 3 In Our DNA SC, Medical University of South Carolina, Charleston, SC, USA; 4 Biomedical Informatics Center, Medical University of South Carolina, Charleston, SC, USA; 5 Department of Neuroscience, Medical University of South Carolina, Charleston, SC, USA

**Keywords:** Consent, recruitment, population screening, large-scale research programs, enhanced consent

## Abstract

The consent process for research studies can be burdensome for potential participants due to complex information and lengthy consent forms. This pragmatic study aimed to improve the consent experience and evaluate its impact on participant decision making, study knowledge, and satisfaction with the In Our DNA SC program, a population-based genomic screening initiative. We compared two consent procedures: standard consent (SC) involving a PDF document and enhanced consent (EC) incorporating a pictograph and true or false questions. Decision-making control, study knowledge, satisfaction, and time to consent were assessed. We analyzed data for 109 individuals who completed the SC and 96 who completed the EC. Results indicated strong decision-making control and high levels of knowledge and satisfaction in both groups. While no significant differences were found between the two groups, the EC experience took longer for participants to complete. Future modifications include incorporating video modules and launching a Spanish version of the consent experience. Overall, this study contributes to the growing literature on consent improvements and highlights the need to assess salient components and explore participant preferences for receiving consent information.

## Introduction

Consent is essential for ethical research, ensuring that participants understand the purpose, risks, and benefits of their involvement in a study. Regulatory requirements and increasingly complex research can result in long, difficult to understand consent forms. Typically, the consent process involves providing written material to participants with limited consideration of how the participant will experience receiving the information or options for alternative approaches to sharing the information.

A growing body of literature has considered approaches to improve the consent experience for research participants, including providing materials in other formats (e.g., visual, auditory, video, and experiential) to help improve experience and comprehension, or providing decision aids [1–3]. Previous research has shown a patient preference for video consents instead of paper consents in some settings, although competency quizzes indicated no significant difference in content comprehension between the two [1]. Another study concluded that video-assisted informed consent could improve satisfaction and comprehension of medical proceedings for trauma patients in the emergency department [2]. Research has also indicated that certain consent procedures may attract more diverse and representative study samples [4]. However, it is widely acknowledged in the field that more research is required to refine current consent recommendations [5].

This paper describes the consent modifications made to enhance the consent experience and whether these enhancements improved participant decision making, study knowledge, and satisfaction consenting to participate in a large-scale population-wide genomic screening program. We compared two consent forms, one with a pictograph and true-false knowledge questions and one without. We sought to identify potential benefits of visual aids and comprehension questions in improving overall consent experience. The two aims of the study were to: 1) describe the consent experience for participants in a large scale population-based genomic screening study and 2) to compare participants decision making, study knowledge, and satisfaction between the standard consent (SC) process and enhanced consent (EC) process.

## Methods

### Setting

Our goal was to optimize the consent experience for individuals interested in the In Our DNA SC program, which is a population-based genomic screening program that began in November 2021 at the Medical University of South Carolina (MUSC). Adult residents of South Carolina are eligible to participate in the program and receive free genetic screening for the Centers for Disease Control and Prevention’s Tier 1 conditions: Hereditary Breast and Ovarian Cancer, Lynch Syndrome, and Familial Hypercholesterolemia. Once the individual provided consent, the individual provides a saliva sample at an upcoming clinical visit, community event, or through an at home sample collection kit. The completed kits are mailed to our industry partner Helix for processing. Participants receive their results via patient portal approximately 8–12 weeks after initial collection.

### Recruitment process for In Our DNA SC

In Our DNA SC’s recruitment strategy encompasses both passive and active approaches, leveraging the power of our website and MUSC’s MyChart for effective outreach. Through our website, potential participants are have access to information about the study and are able to review the consent form. If interested in participating, potential participants are prompted to login to their patient portal to access the consent form. The active recruitment process is facilitated through MyChart recruitment messages which are sent based on upcoming appointments or follow-up direct messages. Potential participants can access the consent form directly from these recruitment and direct messages. Overall, by combining passive and active recruitment strategies, In Our DNA SC attempts to maximize its reach and engagement of potential participants.

### Description of consent process

Individuals who are interested in the In Our DNA SC program complete the informed consent process by reviewing and signing an electronic PDF rendered through a custom web interface that leverages MUSC’s MyChart and REDCap. MUSC’s MyChart houses the consent form and REDCap is used as a backend data source for storing signed versions of the consent form. This consent form is written at an 8^th^ grade reading level and is approximately 17 pages. The electronic consent process verifies that individuals have not already consented for the study by checking their MUSC medical record number against the REDCap database of existing consents. Those who have not consented are able to proceed to the consent form. Potential participants complete the consent process remotely, with the ability to reach the study team via email or phone call if questions arise during the consenting process. The Frequently Asked Questions page on the In Our DNA SC website offer comprehensive responses and can address common inquiries that might arise during the consent process.

**Figure 1.**
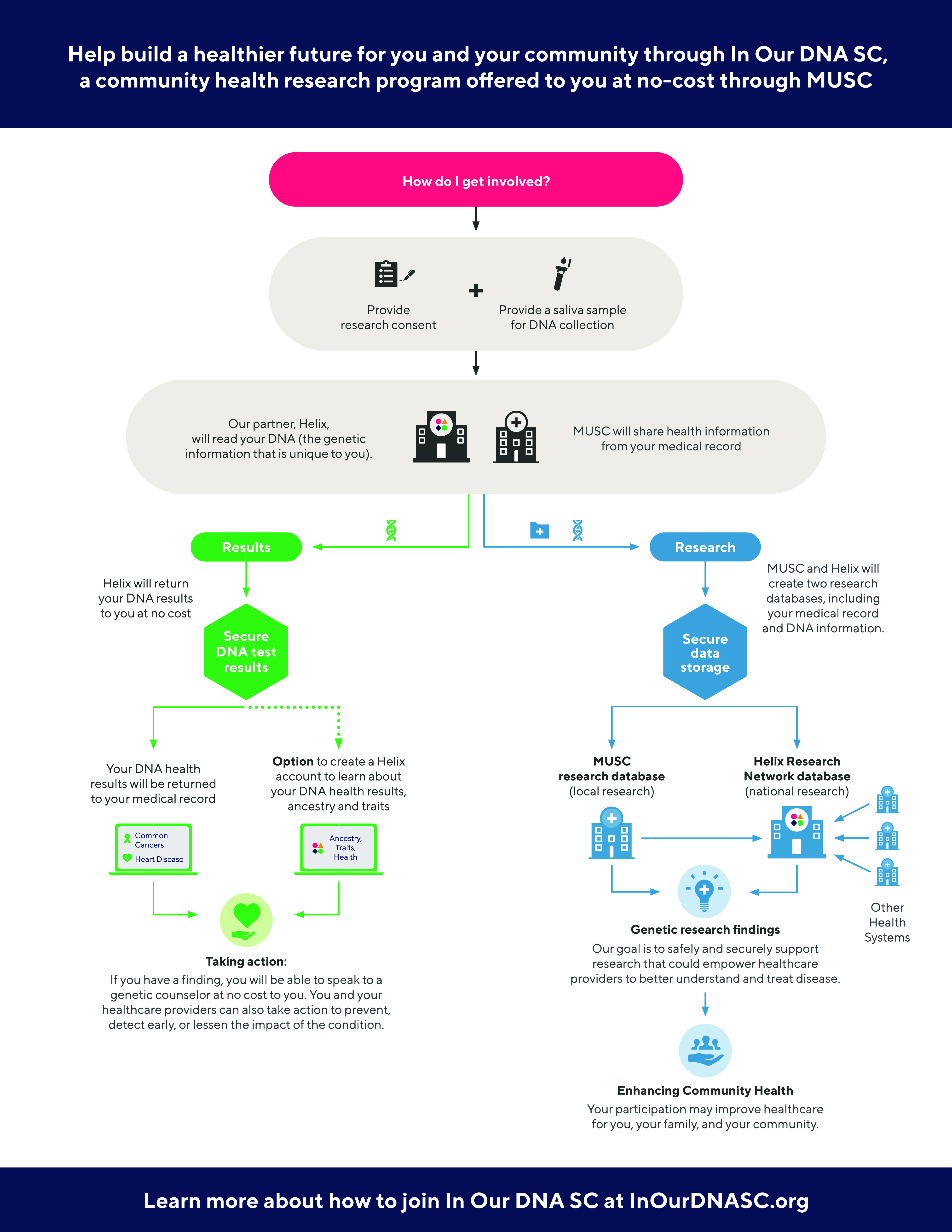
Pictograph used in enhanced consent (EC).

Once consent is captured by REDCap, the individual is enrolled into the program and an order for a DNA sample collection is sent back to their MUSC electronic health record. Two electronic versions of the consent were deployed sequentially: 1) SC, which included a PDF of the consent form (November 8, 2021, through August 9, 2022) and 2) EC, which included a 1-page pictograph outlining the study procedures (Figure 1), PDF of the consent form, and five true-false questions (August 10, 2022, through present).

### Design and data collection

**Figure 2.**
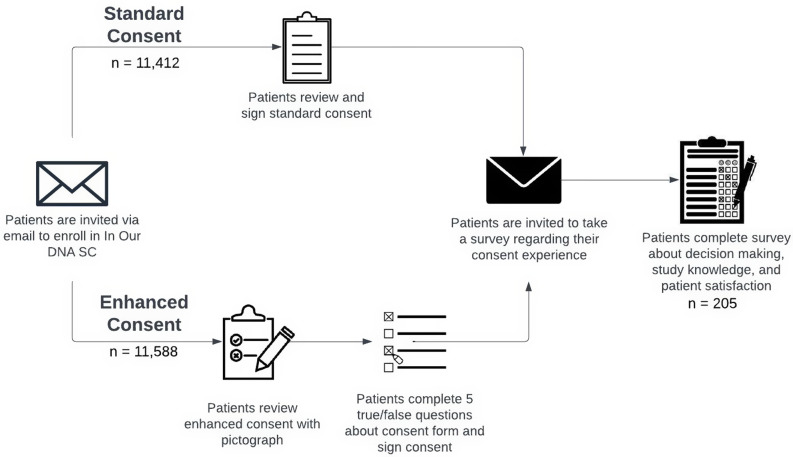
Patient consent experience.

Individuals who consented to participate in the In Our DNA SC program within the previous two weeks were eligible to participate in the post-consent survey assessing decision making, study knowledge, satisfaction, and time to consent between the SC and EC (Figure 2). Participants were offered $5 gift cards in exchange for completing the post-consent questionnaire about their consent experience and were contacted up to three times to recruit for participation.

To assess changes in decision making, we administered the Decision-Making Control Instrument, a validated, 9-item questionnaire (6-point Likert scale ranging from strongly disagree to strongly agree) that asks about level of agreement regarding participant’s perceived decision-making ability to participate in a study. The assessment of decision-making control helps indicate whether the consent materials effectively supported participants in making voluntary and informed decisions, even if they are completing the consent process without direct interactions from the study team. This questionnaire was virtually completed by participants after the completion of their consent process. To assess study knowledge, we asked five questions (5-point Likert scale ranging from strongly agree to strongly disagree) about knowledge using questions adapted from a validated knowledge instrument [6,7]. To assess patient satisfaction, we asked three questions tailored to our specific study (5-point Likert scale ranging from strongly agree to strongly disagree). We assessed time to consent by tracking the amount of time it took for an individual to review the materials, as measured by the time they opened the consent form until the time they submitted it. Finally, we also captured data about participant’s responses to the true-false questions asked as part of the EC.

### Data analysis

We hypothesized that the inclusion of pictographs in consent forms would enhance participants’ comprehension, engagement, and overall consent quality compared to traditional text-based forms. We completed descriptive statistics (mean, standard deviation) for the Decision-Making Control Instrument, study knowledge, satisfaction, and time to consent. We assessed differences in our sample groups using a chi-square test for categorial variables and ttest for age. To compare differences between SC and EC, we conducted a two-tailed, two-sample equal variance *t*-test.

## Results

We assessed the decision making, study knowledge, satisfaction, and time to consent for 109 individuals that completed the SC procedures for In Our DNA SC. A total of 96 individuals completed these surveys for the EC. There were no significant sociodemographic differences across collection type by gender, race, or ethnicity. The age of those in the SC arm was significantly higher (55.5 years) than the age of those in the EC arm (47.7 years) (*p* < 0.0017) (Table 1).

**Table 1.** Sociodemographic differences across consent type

	Standard (*n*= 109)	Enhanced (*n*= 96)	*p*-value*
Female (%)	68.8	64.6	0.5215
Race (%)			0.2613
Black	7.3	8.3	
White	85.3	77.1	
Asian	0.9	2.1	
Other	0.9	6.3	
Missing	5.5	6.3	
Ethnicity (%)			0.7946
Hispanic/Latino	2.8	4.2	
Non-Hispanic/Latino	85.3	82.3	
Missing	11.9	13.5	
Age (mean (sd))	55.5 (17.2)	47.7 (18.1)	0.0017

Overall, individuals felt they had strong Decision-Making Control across both the SC and EC (Table 2). There were no significant differences between SC and EC Decision-Making Control scores. Individuals “strongly disagreed” with the statement “I was powerless in the face of this decision,” “Someone took this decision away from me,” “I was passive in the face of this decision,” “The decision to enroll was inappropriately influenced by others,” “I was not in control of this decision,” “Others made this decision against my wishes,” “I was not the one to choose.” Individuals strongly agreed with the following statements, “I made this decision,” and “The decision was up to me.”

**Table 2.** Comparing Standard Consent and Enhanced Consent decision making, study knowledge, and patient satisfaction

	Standard Consent (SC)		Enhanced Consent (EC)		
	Mean	Standard Deviation	Mean	Standard Deviation	*p*-value
**Decision-making scale**					
I was powerless in the face of this decision	1.06	0.27	1.19	0.65	0.054
Someone took this decision away from me	1.02	0.13	1.07	0.26	0.055
I made this decision	5.82	0.72	5.95	0.22	0.088
I was passive in the face of this decision	1.75	1.50	1.74	1.42	0.951
The decision to enroll was inappropriately influenced by others	1.15	0.70	1.06	0.24	0.266
I was not in control of this decision	1.23	0.95	1.04	0.20	0.061
Others made this decision against my wishes	1.05	0.21	1.05	0.22	0.838
I was not the one to choose	1.09	0.52	1.04	0.20	0.375
The decision was up to me	5.81	0.74	5.86	0.57	0.540
**Study knowledge**					
I know what happens during the study, how long it will last, and what I am being asked to do	4.17	0.93	4.29	0.87	0.344
I know what the study will do with my DNA samples and my information	4.33	0.80	4.34	0.86	0.908
I know whether my health insurance will be impacted by participating in the study	3.97	1.05	4.08	1.12	0.466
I know what health-related results I will get form participating in the study	4.08	0.88	4.44	0.69	0.002
I know how my privacy and confidentiality will be protected	4.39	0.80	4.48	0.78	0.399
**Patient satisfaction**					
I can comprehend the information that was shared as part of the consent process for In Our DNA SC	4.57	0.67	4.58	0.64	0.875
The information in the consent form will help me make informed decisions about participation in the In Our DNA SC program	4.53	0.62	4.53	0.74	0.993
I am satisfied with the consent process of In Our DNA SC	4.55	0.63	4.58	0.64	0.713

Individuals also had high levels of knowledge across both SC and EC forms. The lowest understanding reported was understanding of the impact that participating in the program has on one’s health insurance. There was a significant increase in understanding of what health-related results the participant would get from the study between SC and EC.

Patient satisfaction was rated highly for both SC and EC forms, with no significant differences across satisfaction scales.

Average time to consent for all who completed SC was 5:07, and all who completed EC was 11:29. We found high understanding based on responses to true-false questions, which were provided to EC participants (*n*= 11,588) (Table 3). Overall, participants answered most questions correctly. The lowest scoring item was “Once, I enroll in the study, I cannot withdraw” (86.8% correct), followed by, “I do not need to sign a research consent to participate” (87.3% correct), “If I receive a DNA test result from the study that impact my health, I will be able to speak to a genetic counselor at no cost” (92.8% correct), “You will receive results about inherited risks for certain types of cancer and heart disease. These results will go into your medical records” (93.7% correct), and “If you participate your DNA will be securely stored and used for approved research” (98.9% correct).

**Table 3.** Enhanced consent true-false questions

Question	Number correct	% Correct
I do not need to sign a research consent to participate (False)	10,115	87.3
If I receive a DNA test results from the study that impacts my health, I will be able to speak to a genetic counselor at no cost (True)	10,758	92.8
Once I enroll in the study, I cannot withdraw (False)	10,061	86.8
If you participate your DNA will be securely stored and used for approved research (True)	11,456	98.9
You will receive results about inherited risks for certain types of cancer and heart disease. These results will go into your medical record (True)	10,856	93.7
	Combined (*N*= 11,588)	

## Discussion and conclusions

We compared SC and EC among individuals who participated in a population-wide genomic screening study. Apart from, “I know what health -related results I will get from articipating in this study,” we did not find significant differences in In Our DNA SC participant’s perceived decision making, study knowledge, or satisfaction between SC and EC. The significant increase in understanding of what health-related results the participant would get from the study between SC and EC could have been attributed to the true-false questions in the EC which may have reinforced key information. However, the general lack of significant differences between the results for EC and SC indicate that our hypothesis that the inclusion of pictographs in the consent form would enhance participants comprehension, engagement, and overall consent quality was not fully supported by the results.

Possible reasons for the lack of differences between SC and EC may include that the study has a companion website, which has substantial information about the program, including a series of frequently asked questions. Thus, individuals were may have already reviewed the website materials and had a clear understanding of the study and their decision to participate. Further, it is notable that MUSC’s standard research recruitment strategy applies an opt-out approach [8]. Therefore, patients are eligible to be contacted via MyChart for research recruitment unless they ask to be excluded, which can be done in multiple ways. Studies indicate an opt-out approach have been shown to increase patient access to research participation and increase the likelihood of more representative study samples when compared to opt-in methods, with no significant difference in complaints or distress [9]. This opt-out approach may have contributed to a higher likelihood of individuals knowing about the study or about research in general. Furthermore, although the scales used in this study were validated instruments, these there were consistently high scores, which may indicate a ceiling effect and limit the ability to detect differences between consent forms. Future research could consider using more sensitive scales or alternative assessment methods to capture a larger range of participant experiences and identify more nuanced areas for improvement.

Both the SC and EC groups indicated comprehension of the study requirements, suggesting that participants in both groups had access to sufficient educational materials to complete the consent. While we focused on the average differences in consent for SC and EC, future research may be interested in preventing very low scores in these domains. It is noteworthy that EC participants took longer to complete the consent, which researchers hypothesize may correlate with increased comprehension or satisfaction. A future study may include comprehension questions and satisfaction questions for both SC and EC participants, to compare with self-reported assessments of comprehension, and indicate more thoroughly if differences between the two consents may be present. For example, prior research in different specialties demonstrated patient preference for visual consents, so evaluating patient consent preferences in population genetic screening programs would add to the existing literature [1].

Additionally, researchers could explore how different educational approaches, such as video presentations or interactive tools, impact participant comprehension and decision making during the consent process [2]. There are many opportunities to research improving pictographs as part of the consent experience, such as surveys to directly evaluate participants’ perceptions of the pictographs. Furthermore, investigating the long-term impact of comprehension on their experience and satisfaction with the research could enhance our understanding of overall involvement in genomic studies. Prior research has investigated correlations between consent procedures and diversity and representation in genomics research [4]. Another important metric to collect in future studies is percentage and demographic data on individuals who started the consent process and did not complete it. Some groups may be less likely to complete the consent once started, which is important for promoting diversity and inclusion in the consent process and genomics research broadly.

One limitation for analysis was that we did not have true-false questions directly measuring content understanding for the EC to compare to the true-false data from the SC. Although overall comprehension was high in the EC group, due to the pragmatic nature of this study, we did not have true-false data available in the SC group. Instead, we compared the study knowledge between the consent forms through self-reported metrics. Participants may have been overconfident when indicating their understanding of the consent form on the questionnaire. Additionally, adding in true-false questions to the SC would have undermined the quality of the study because the consent form would no longer qualify as a SC. Future studies may also investigate study knowledge between multiple consent forms directly through asking true-false content questions for the SC. Another consideration is that this study was not randomized due to feasibility constraints, so an observational approach was adopted. One opportunity to expand upon this work in the future would be to iterate a similar study as a randomized control trial. Another limitation is that the number of individuals who completed these consent forms for the study was evaluated, but the number of individuals who started the consent form and failed to complete it was unable to be captured. These data would be a relevant indicator of patient experience and preference and should be evaluated in future studies.

Additional modifications are planned to the In Our DNA SC consent experience. This includes the development of ten video modules that are designed to highlight and expand upon key components of the consent. These videos will be embedded as part of the consent form and participants will have the option of viewing them. We also plan to launch a Spanish version of the consent experience, including translation of the study website, pictograph, consent form, true-false questions, and videos. Additional modifications to the pictograph, including more details about the risks that may be associated with participation, could be included.

Our findings build upon prior literature seeking to improve the consent experience by providing various formats for participants to ingest consent information. While we did not find significant differences in SC or EC, our approach was embedded in an existing, ongoing study. Thus, we did not randomize individuals to a specific consent experience. Given that individuals had similar experience regardless of SC or EC, further assessment could consider which components of the consent are most salient, provide potential participants with a choice about the way they wish to receive consent information (e.g., visual and audio), or explore how information participants receive prior to the consent (e.g., website materials and frequently asked questions) impact the consent experience.
